# Eyes-Open Fronto-Central Alpha Activity as an Electrophysiological Correlate of Working Memory in Children with Predominantly Inattentive ADHD

**DOI:** 10.3390/diagnostics16193104

**Published:** 2026-09-24

**Authors:** Chih-Hao Wang, Yu-Hsin Huang, Yun-Tse Lee, Hao-Wei Wang, Yi-Hung Liu, Hui-Ju Chen

**Affiliations:** 1 Department of Pediatrics, MacKay Children’s Hospital, Taipei 104217, Taiwan; mmh4972@gmail.com; 2 Division of Psychiatry, MacKay Memorial Hospital, Taipei 104217, Taiwan; phoebe.huang@gmail.com (Y.-H.H.); ficlwa@yahoo.com.tw (Y.-T.L.); 3 School of Medicine, College of Medicine, MacKay Medical University, New Taipei City 252005, Taiwan; 4 Department of Audiology and Speech-Language Pathology, MacKay Medical University, New Taipei City 252005, Taiwan; 5 Department of Child and Adolescent Psychiatry, Tsaotun Psychiatric Center, Ministry of Health and Welfare, Nantou 542019, Taiwan; ghowdayway@gmail.com; 6 The Institute of Electrical and Control Engineering, National Yang Ming Chiao Tung University, Hsinchu 300093, Taiwan; 7 Child Developmental Assessment & Intervention Center, MacKay Memorial Hospital, Taipei 104217, Taiwan; 8 Department of Pediatric Neurology, MacKay Children’s Hospital, Taipei 104217, Taiwan

**Keywords:** ADHD, QEEG, alpha oscillations, working memory, resting-state EEG

## Abstract

**Background/Objectives**: This study aimed to identify resting-state quantitative electroencephalography (QEEG) correlates of cognitive performance in children with attention-deficit/hyperactivity disorder (ADHD). **Methods**: ADHD diagnoses were established according to the Diagnostic and Statistical Manual of Mental Disorders, Fifth Edition. Cognitive performance was evaluated using the Wechsler Intelligence Scale for Children, Fourth Edition, focusing on the Working Memory Index (WMI) and Processing Speed Index (PSI). Following at least 24 h medication washout, QEEG recordings were collected during both the eyes-open (EO) and eyes-closed (EC) resting states. The absolute spectral power across four frequency bands (delta, theta, alpha, and beta) was analyzed within seven predefined scalp regions. Associations between QEEG measures and cognitive indices were assessed using Spearman’s rank correlation with false discovery rate (FDR) correction. **Results**: A total of 45 children with ADHD were included: 29 with predominantly inattentive ADHD (ADHD-I) and 16 with combined ADHD (ADHD-C). No significant associations between the QEEG measures and cognitive performance were observed in the overall ADHD cohort. However, within the ADHD-I presentation, EO alpha power was positively correlated with WMI scores across bilateral frontal and central regions (Spearman’s *ρ* = 0.535–0.582, FDR-corrected *q* = 0.014–0.028). In contrast, no significant alpha–WMI associations were identified in the ADHD-C presentation. Furthermore, no significant correlations were observed during the EC condition, and no EEG-PSI associations survived the FDR in either presentation. **Conclusions**: Our findings demonstrate presentation-related differences in QEEG–cognitive relationships and suggest that EO frontocentral alpha activity may represent an electrophysiological correlate of working memory performance in pediatric ADHD-I, though limited by the lack of healthy controls.

## 1. Introduction

Attention-deficit/hyperactivity disorder (ADHD) is a common neurodevelopmental disorder in children characterized by symptoms of inattention, hyperactivity, and impulsivity [1]. The prevalence of ADHD remains substantial globally, with approximately 11.4% of children aged 3–17 years having received a diagnosis in the United States [2], while the estimated prevalence in Taiwan is about 10.1% [3]. Beyond behavioral manifestations, cognitive dysfunction is increasingly being recognized as a major contributor to adverse functional outcomes in ADHD. Among these impairments, deficits in working memory and processing speed are among the most consistently reported cognitive abnormalities [4,5]. Such deficits compromise the child’s ability to maintain, manipulate, and efficiently process information, thereby negatively affecting academic achievement, social interactions, and daily functioning [4,5,6,7]. Therefore, early and accurate assessment of cognitive function is critical for children with ADHD to facilitate timely intervention and reduce long-term functional impairment [4,5].

Currently, the assessment of cognitive function in children with ADHD relies largely on standardized neuropsychological instruments, such as the Wechsler Intelligence Scale for Children (WISC) [4,6]. Although these tools provide comprehensive cognitive profiling, their administration is time-consuming, typically requiring 1.5–2 h per child, and depends on trained psychologists for administration and interpretation [8,9]. In many clinical settings, particularly in public healthcare systems, these requirements contribute to prolonged waiting times and delayed access to intervention services [8]. Consequently, there is a growing need for objective, rapid, and readily accessible tools that complement conventional neuropsychological assessments [10,11,12].

Quantitative electroencephalography (QEEG) has emerged as a promising tool for investigating neural mechanisms underlying cognitive dysfunction in ADHD. By providing objective measures of brain activity, QEEG may facilitate the identification of neurophysiological correlates of cognitive function [10,13,14]. Spontaneous oscillatory activity across different EEG frequency bands has been linked to fundamental neurocognitive processes, including attention, executive control, working memory, and information processing [15,16,17]. Consequently, the characteristic EEG abnormalities observed in children with ADHD, including elevated slow-wave activity (delta and theta), altered alpha oscillations, and atypical beta activity, are increasingly viewed as electrophysiological markers of the disorder itself and as potential indicators of the neural dysfunction underlying ADHD-related cognitive impairments [12,18,19].

However, the existing literature remains heterogeneous and inconclusive [18]. These reported associations vary considerably across EEG frequency bands, brain regions, cognitive domains, and study populations [19]. In particular, the influence of resting-state conditions, such as eyes-open (EO) and eyes-closed (EC) recordings, has received relatively limited attention, despite evidence that these states reflect distinct neural network configurations, such as differential modulation of the default mode network, and varying levels of cortical alertness and cognitive processes [20,21]. Consequently, the relationship between different resting-state QEEG conditions (EO vs. EC) and specific cognitive functions in children with ADHD remains unclear.

Therefore, this study aimed to investigate the association between resting-state QEEG activity and cognitive function in children with ADHD under both EO and EC conditions. By examining QEEG measurements obtained under different resting-state conditions, we sought to determine whether distinct neurophysiological patterns were associated with cognitive performance. Given the reported neurophysiological heterogeneity across ADHD presentations, potential presentation-related differences were also explored. We hypothesized that resting-state QEEG features would be associated with cognitive functioning, while presentation- and condition-related differences were examined exploratorily.

## 2. Materials and Methods

### 2.1. Study Design and Participants

This study was conducted at the Pediatric Neurology and Psychiatry Outpatient Clinic of MacKay Memorial Hospital between 2019 and 2021, recruiting children aged 8 to 15 years. ADHD diagnoses were independently evaluated by two board-certified physicians based on the Diagnostic and Statistical Manual of Mental Disorders, Fifth Edition (DSM-5) criteria. Inter-rater reliability was excellent for both the predominantly inattentive (Cohen’s *κ* = 0.93) and combined (Cohen’s *κ* = 0.87) presentations. Finally, the diagnosis and specific presentation were confirmed by a child psychiatrist using the Kiddie Schedule for Affective Disorders and Schizophrenia—Epidemiologic Version (K-SADS-E).

Participants were excluded if they met any of the following criteria: (1) an intelligence quotient (IQ) < 85; (2) a score ≥ 15 on the Childhood Autism Spectrum Test (CAST), or meeting the criteria for autism spectrum disorder; (3) a history of neurological or systemic illness, or major psychiatric disorders (e.g., major depressive disorder, bipolar disorder, schizophrenia, severe anxiety disorders such as panic disorder, and obsessive–compulsive disorder); (4) learning disabilities; (5) a history of substance use disorder in the previous 6 months; or (6) usage of central nervous system (CNS) stimulants within 24 h prior to the assessment.

We also collected cognitive data using the Wechsler Intelligence Scale for Children, Fourth Edition (WISC-IV; Traditional Chinese revised edition). This study focused on the Working Memory Index (WMI) and Processing Speed Index (PSI) a priori, as these domains are among the most consistently reported cognitive deficits in children with ADHD and are closely related to attentional control and executive functioning. Conversely, the Verbal Comprehension Index and Perceptual Reasoning Index were excluded because they represent general cognitive abilities typically preserved in ADHD, rather than core executive vulnerabilities [4]. The WMI was calculated using the Digit Span and Letter-Number Sequencing core subtests, while the PSI was derived from the Coding and Symbol Search core subtests. WISC-IV assessments and QEEG analyses were conducted independently, within two weeks of each other, by separate experts who were mutually blinded to each other’s findings.

Furthermore, a complete-case analysis approach was adopted. Only participants with artifact-free QEEG recordings and complete neurocognitive assessments were included in the final analysis.

The study was approved by the Institutional Review Board (IRB) of MacKay Memorial Hospital (approval number: 18MMHIS071e). After the aims of this study were fully explained, written informed consent was obtained from the participants’ parents or legal guardians. In addition, participating children provided written assent by signing an IRB-approved, age-appropriate assent form.

### 2.2. EEG Recording and Preprocessing

Participants were instructed to wash their hair prior to the recording session to minimize scalp impedance and improve signal quality. The participants were seated comfortably in a chair with armrests positioned approximately 70 cm from the LCD screen. The room was kept quiet, and nonessential electronic devices were turned off. The experiment was explained in detail to the participants to ensure compliance. All participants were medication-free for at least 24 h before the session.

EEG signals were recorded using a 33-channel electro-cap connected to a 40-channel amplifier (NuAmps, Compumedics, Charlotte, NC, USA). Ag/AgCl electrodes were positioned according to the international 10–20 system at 19 scalp locations (Fp1, Fp2, Fz, F3, F4, F7, F8, Cz, C3, C4, T3, T4, T5, T6, Pz, P3, P4, O1, and O2), with Cz used as the anatomical landmark for cap positioning. Prior to electrode placement, skin preparation included exfoliation and alcohol-based cleaning. The signal quality of all EEG channels was carefully monitored during data acquisition, and the electrode–skin impedance was maintained below 10 kΩ using conductive electrode gel. No channels exhibited persistent excessive noise, signal saturation, or poor electrode contact; therefore, no bad channels were identified or excluded from subsequent analysis.

The ground electrode was placed on the forehead and also used as the physical reference electrode during recording. Electrodes placed lateral to the right eye and above the left eye were used to monitor electrooculography (EOG) signals. EEG and EOG signals were amplified, online band-pass filtered between 0.5 and 100 Hz using a Neuroscan amplifier, and digitized at a sampling frequency of 500 Hz. A 60-Hz notch filter was applied to suppress power-line interference. No offline filtering was performed, as the 0.5-Hz online high-pass filter sufficiently eliminated low-frequency drifts.

Resting-state EEG data were recorded in two separate rounds. Each round comprised a 150 s EO period (fixation on a black cross), a 15 s transition break, a 150 s EC period, and a subsequent 15 s transition break (Figure 1A). The recorded EEG signals were subsequently re-referenced offline to the average of bilateral earlobes. EOG-related artifacts were first removed using the artifact-removal tool implemented in Scan 4.5 (NeuroScan). Independent component analysis (ICA; Infomax implemented in EEGLAB [22]) in conjunction with the ADJUST algorithm [23] was subsequently applied to further identify and remove artifact-related components, including those associated with eye blinks, eye movements, and electromyographic (EMG) activity. ADJUST has been widely used for automatic identification of artifact-related independent components (e.g., [24]). The artifact-corrected EEG signals were then used for all subsequent analyses. Participants were continuously observed by the EEG technician during recording to ensure that they remained awake throughout each 150 s block.

### 2.3. Power Spectral Analysis of the EEG Recordings

Prior to spectral analysis, raw EEG data underwent rigorous trimming and epoching procedures. To minimize transition-related artifacts and improve signal stability, the first and last 15 s of each 150 s recording block were discarded. This procedure resulted in 120 s of high-quality data per block, yielding 240 s of retained data for each condition (EO and EC). The retained data were segmented into 30 non-overlapping, artifact-corrected epochs, each 8 s long, with all 30 epochs fully analyzed per participant after Scan 4.5 and ICA-ADJUST correction. Each 8 s EEG epoch (4000 samples) was multiplied by a Hamming window prior to spectral analysis to reduce spectral leakage. The fast Fourier transform (FFT) was then applied to each windowed epoch, and the power at each frequency bin was calculated as the squared magnitude of the corresponding FFT coefficient. No additional normalization was applied to the FFT coefficients. The band power (BP) was calculated by summing the power values across all frequency bins within each frequency band. Four frequency bands were examined: delta (1–4 Hz), theta (4–8 Hz), alpha (8–13 Hz), and beta (13–30 Hz). For each EEG channel, BP was calculated separately for each epoch and then averaged across all 30 epochs. The resulting averaged BP was defined as the absolute power of the corresponding frequency band, without normalization to the total spectral power (Figure 1B).

Absolute spectral power was analyzed instead of relative power in consideration of the neurophysiological heterogeneity of ADHD. A commonly reported electrophysiological pattern in children with ADHD is increased slow-wave activity accompanied by reduced fast-wave activity [12,18]. Because relative power values are mathematically interdependent, prominent slow-wave elevations may proportionally reduce relative power values in other frequency bands [25]. Therefore, absolute power was selected to characterize band-specific spectral activity without this proportional dependence.

All electrophysiological and clinical data were de-identified and stored in a secure, access-controlled institutional system accessible only to authorized study personnel.

### 2.4. Definition of Regions of Interest (ROIs)

EEG electrodes were grouped into seven ROIs based on their anatomical locations, i.e., left frontal (LF: F7, Fp1, and F3), right frontal (RF: Fp2, F4, and F8), central (CN: C3, Cz, and C4), left temporal (LT: T3, and T5), right temporal (RT: T4, and T6), left parieto-occipital (LPO: P3, and O1), and right parieto-occipital (RPO: P4 and O2), to facilitate clinical interpretation and spatial analysis (Figure 2). The midline electrodes Fz and Pz were excluded from the ROI grouping to focus on lateralized and primary regional differences.

For each ROI, absolute band power was calculated by averaging the power values of the corresponding electrodes. For example, CN power was computed as the mean of C3, Cz, and C4.

### 2.5. Statistical Analysis

Normality of the data distribution was assessed using the Shapiro–Wilk test. Continuous demographic and clinical variables are presented as mean ± standard deviation (SD) and were compared between the ADHD-I and ADHD-C presentations using the independent-samples *t*-test. Categorical variables are presented as counts (percentages) and compared using the chi-square test or Fisher’s exact test, as appropriate.

To evaluate the relationship between QEEG absolute power and targeted WISC-IV cognitive indices (WMI and PSI), the non-parametric Spearman’s rank correlation coefficient (*ρ*) was utilized, as the QEEG spectral power data did not strictly conform to a normal distribution. Bootstrap 95% confidence intervals (CI; 1000 resamples) were calculated for Spearman’s *ρ*. Crucially, to rigorously control for false-positive findings (Type I errors) during the regional and frequency-band analyses, the Benjamini–Hochberg procedure was applied to control the false discovery rate (FDR). For each cognitive outcome (WMI and PSI), FDR correction was applied across a predefined family of 28 comparisons (4 frequency bands × 7 ROIs). These within-family FDR corrections did not constitute a global correction across all combinations of cognitive outcomes, recording conditions, and diagnostic presentations; accordingly, subgroup findings were interpreted as exploratory.

To evaluate potential confounding effects of developmental stage and biological sex, multivariable linear regression analyses simultaneously adjusting for age and sex were conducted as sensitivity analyses. Univariate General Linear Models (GLMs) evaluated the Presentation (ADHD-I vs. ADHD-C) × WMI interaction on EO alpha power across frontocentral ROIs, estimating slope differences (Δ*B* [95% CI]) relative to ADHD-C. Repeated-measures GLMs incorporating both EO and EC conditions were additionally used to evaluate WMI × State and WMI × Presentation × State interactions.

All statistical analyses were conducted using IBM SPSS Statistics (version 26.0; IBM Corp., Armonk, NY, USA) and Python (version 3.10; Python Software Foundation, Wilmington, DE, USA). A two-tailed *p*-value < 0.05 and an FDR-adjusted *q*-value < 0.05 were considered statistically significant.

## 3. Results

### 3.1. Demographic and Clinical Characteristics

A total of 83 children were initially recruited for the study. Of these, 38 were excluded from the final analysis: Eleven did not undergo QEEG recording, twenty-four did not complete the WISC-IV assessment, two did not meet the diagnostic criteria for ADHD based on the K-SADS-E, and one had an IQ < 85. The final analysis therefore included 45 children with ADHD, comprising 29 with ADHD-I and 16 with ADHD-C. Demographic characteristics were comparable between the two presentations, with no significant differences in age, sex distribution, body mass index (BMI), gestational age, or birth weight (all *p* > 0.05). Similarly, cognitive profiles, including Full-Scale IQ, WMI, and PSI, did not differ significantly between the ADHD-I and ADHD-C groups (Table 1).

### 3.2. Association Between QEEG Measures and WMI

No significant correlations were observed between QEEG and WMI scores in the overall ADHD cohort. However, within the ADHD-I presentation, EO alpha power demonstrated a localized fronto-central pattern of positive correlations with WMI scores, involving the CN, RF, and LF regions (Spearman’s *ρ* = 0.535–0.582; Figure 3). These associations remained significant even after false discovery rate (FDR) correction (Table 2).

The robustness of these findings was further supported by sensitivity analyses. Exclusion of potential high-power outliers (alpha power > 10 μV^2^ in CN, *n* = 2; >5 μV^2^ in RF/LF, *n* = 1; as plotted in Figure 3A–C) yielded consistent positive correlations (CN: *ρ* = 0.532, *p* = 0.004; RF: *ρ* = 0.549, *p* = 0.002; LF: *ρ* = 0.516, *p* = 0.005). Similarly, multivariable linear regression analyses adjusting simultaneously for participant age and sex confirmed that WMI remained significantly associated with EO alpha power in the CN (*β* = 0.506, *p* = 0.010), RF (*β* = 0.488, *p* = 0.014), and LF (*β* = 0.466, *p* = 0.020) regions, suggesting that age and sex had limited influence on the observed relationships.

In contrast, no significant alpha–WMI associations were identified in the ADHD-C presentation. Formal GLM analyses further demonstrated significant Presentation × WMI interactions for EO alpha power in the CN, RF, and LF regions (*F*(1, 41) = 4.790–8.314, *p* = 0.006–0.035), indicating that the positive WMI–alpha relationship was significantly stronger in ADHD-I than in ADHD-C (Δ*B* = 0.043–0.148; Table 3).

Furthermore, no significant correlation was observed during the EC in any group or subgroup. Formal interaction analyses incorporating both states did not demonstrate significant state-related differences in these associations. Detailed alpha-band correlation matrices are presented in Supplementary Table S1, and comprehensive results for the delta, theta, and beta frequency bands are provided in Supplementary Table S3.

### 3.3. Correlations Between QEEG Measures and PSI

No significant associations were identified between alpha power and PSI scores across either the EO or EC conditions in the overall ADHD cohort or ADHD presentation. The detailed alpha-band results are presented in Supplementary Table S2. The comprehensive correlation matrices for the delta, theta, and beta frequency bands are provided in Supplementary Table S4.

## 4. Discussion

### 4.1. Summary of Primary Findings

Our findings revealed presentation-related differences in the relationship between resting-state QEEG activity and cognitive performance, whereby alpha power during the EO condition, but not during the EC condition, was positively associated with WMI in the ADHD-I presentation. These associations were observed across the central and bilateral frontal regions, demonstrated large effect sizes, and remained significant after FDR correction. In contrast, no significant association was identified between the alpha power and PSI. Collectively, these findings suggest that EO resting-state alpha activity may represent an electrophysiological correlate of working memory performance in children with ADHD, particularly those with an inattentive presentation.

### 4.2. Alpha Oscillations and Working Memory Function

Historically, alpha-band oscillations were considered to reflect a state of “cortical idling.” However, contemporary models increasingly recognize alpha activity as an active top-down inhibitory mechanism that plays a central role in cognitive control [26,27,28]. According to the inhibition timing hypothesis, alpha oscillations suppress task-irrelevant information and protect task-relevant representations from external interference [25,29]. This inhibitory control is fundamental to working memory, which requires maintaining and manipulating information while resisting distraction. Consistent with this framework, higher alpha power has been associated with a more efficient allocation of cognitive resources, greater resistance to distractors, and superior working memory performance [26,30]. In addition, resting-state alpha activity has been proposed to reflect an individual’s trait-like capacity for proactive cognitive control [31]. Crucially, recent network analyses in ADHD populations have demonstrated that executive functioning acts as a critical “bridge node” connecting alpha oscillations to behavioral symptoms, underscoring that alpha activity exerts its clinical influence primarily through higher-order cognitive networks, including working memory [32]. Therefore, the positive association between alpha power and WMI observed in the present study is consistent with, but does not directly demonstrate, theoretical models linking alpha activity to top-down inhibitory control and working memory function.

### 4.3. Fronto-Central Alpha Activity and Executive Control

The observed associations were detected over bilateral frontal and central scalp regions. Although alpha oscillations have traditionally been associated with posterior sensory processing, accumulating evidence indicates that frontocentral alpha activity also plays a critical role in higher-order cognitive control. Working memory relies heavily on top-down regulation coordinated by the prefrontal cortex. Frontocentral alpha activity has been implicated in maintaining task-relevant representations and suppressing distracting information [33,34,35]. Moreover, individual differences have been linked to greater prefrontal alpha activity and superior working memory capacity and cognitive control [36], while neuromodulation experiments have demonstrated that targeted stimulation of prefrontal regions can alter frontocentral neural dynamics and improve working memory performance [37]. Taken together, these findings suggest that resting-state frontocentral alpha activity may reflect neural processes relevant to executive control and working memory function.

### 4.4. Presentation-Related Differences: Why ADHD-I?

The presentation-related differences in the observed associations highlight the neurobiological heterogeneity of ADHD and support the notion that distinct ADHD presentations may involve different cognitive and electrophysiological mechanisms [10,12,38]. This presentation-related association is formally supported by the significant Presentation × WMI interactions on EO alpha power across frontocentral regions, reinforcing the neurobiological distinction between these phenotypes. ADHD-I is characterized by prominent inattentive symptoms, including distractibility, impaired attentional control, and difficulty in maintaining goal-directed attention. Previous electrophysiological evidence suggests that this phenotype is associated with impairments in alpha-mediated sensory gating and visual cue processing [11]. Consequently, disruptions in alpha-related mechanisms may have a more direct impact on cognitive performance in this presentation.

In contrast, children with ADHD-C exhibit additional deficits related to hyperactivity, impulsivity, motor preparation, and response inhibition. Neurophysiological studies have suggested that these symptoms involve a broad range of neural processes, including abnormalities in motor-related beta activity [11]. Indeed, comprehensive reviews have explicitly indicated that aberrant alpha modulation may be more prominent in ADHD-I than in ADHD-C [39]. Taken together, these findings suggest that alpha activity may be particularly relevant to the inattentive dimension of ADHD and are consistent with the possibility that distinct neurophysiological mechanisms may contribute to different ADHD presentations.

### 4.5. Eyes-Open Alpha–WMI Association: Why EO?

Finally, the alpha–WMI association was observed during the EO condition but not during EC recordings, although formal interaction analyses did not establish a significant difference between states. Consistent with previous models, the EC state was characterized by high global alpha synchrony, reflecting a baseline mode of reduced sensory engagement and lower cortical arousal [40]. In contrast, EO recordings involve unstructured visual processing of the immediate environment and represent a more controlled cognitive condition, resulting in a distinct neurophysiological profile characterized by increased cortical arousal and more organized network activation [21,40]. Importantly, recent studies have demonstrated that the relationship between spontaneous alpha activity and proactive cognitive control is most evident during EO conditions. In contrast, EC-alpha activity is less strongly associated with these cognitive processes [31]. This is particularly relevant, given that children with ADHD exhibit a significant atypical reduction in relative alpha power, specifically during the EC state [20], potentially reducing the sensitivity of EC recordings for detecting cognition-related individual differences. These observations provide a possible context for why the alpha–WMI association was detected during EO but not EC in the present sample; however, the absence of a significant state interaction precludes concluding that the association is EO-specific. Together with the presentation differences discussed above, these findings support further investigation of resting-state condition as a potential moderator of QEEG–cognition relationships in larger cohorts.

### 4.6. Limitations and Future Directions

This study has several limitations that should be considered when interpreting the findings. First, the study lacked a typically developing (TD) healthy control group. Although our primary aim was to investigate QEEG–cognition relationships within the ADHD population, the absence of a neurotypical baseline precluded us from determining whether the observed resting-state alpha–WMI association was an ADHD-specific neurophysiological feature or a general developmental mechanism shared across all children. Future studies incorporating a TD control group are required to clarify the disease specificity of these QEEG–cognition associations.

Second, the overall sample size, particularly within ADHD presentations, was relatively modest, and no a priori power analysis was performed. Nevertheless, the primary findings in the ADHD-I presentation demonstrated large effect sizes and remained significant after FDR correction. However, subgroup-level analyses may have been underpowered to detect weaker or subtle neurophysiological associations, particularly within the ADHD-C presentation. Accordingly, nonsignificant demographic differences between ADHD presentations should not be interpreted as evidence of group equivalence. The limited sample size was partially attributable to recruitment challenges during the COVID-19 pandemic, which reduced the number of clinical visits and delayed participant enrollment. Moreover, the single-center sampling may limit the representativeness and generalizability of the study population. Furthermore, none of the participants met the criteria for predominantly hyperactive/impulsive presentation (ADHD-HI), precluding analysis across the full ADHD diagnostic spectrum.

Third, given the cross-sectional and exploratory nature of this study, our results should be interpreted with caution, and causal relationships between QEEG brain dynamics and cognitive performance cannot be established. In addition, ADHD symptom severity was not incorporated as an analytical covariate; therefore, residual confounding by clinical severity cannot be completely excluded. Future longitudinal studies are warranted to elucidate temporal associations between electrophysiological maturation and cognitive development.

Fourth, this study exclusively utilized resting-state QEEG data. All electrophysiological data were collected under nontask conditions (EO and EC). Consequently, these resting-state findings may not be directly generalizable to active cognitive states, and future research incorporating task-based EEG paradigms is recommended. Additionally, while a fixed canonical alpha band (8–13 Hz) was utilized in the present study, future research incorporating individual alpha frequency (IAF) tuning may further clarify subtle developmental variations.

Fifth, the current cohort was predominantly male (80.0%). While this sex distribution accurately reflects the well-documented male predominance in clinical ADHD populations, it limits the generalizability of our findings to female children with ADHD, who may exhibit distinct neurodevelopmental and electrophysiological trajectories.

Sixth, WMI is not a process-pure measure of working memory, and the absence of significant PSI associations does not establish cognitive specificity. Future studies incorporating broader cognitive measures are needed to determine whether the observed alpha–WMI association reflects working-memory-related or more general cognitive processes.

Seventh, EO consistently preceded EC in both recording rounds; therefore, resting-state condition and within-round recording order could not be independently disentangled. Potential habituation, fatigue, or adaptation effects associated with the fixed recording sequence cannot be excluded, and future studies should employ randomized or counterbalanced EO/EC sequences.

Finally, although ADHD medications were withheld for at least 24 h before EEG acquisition, this duration may have been insufficient to eliminate the residual pharmacological effects on brain oscillatory dynamics. Detailed patient medication history and estimated pharmacokinetic clearance profiles are presented in Supplementary Table S5. Despite these limitations, a strength of the present study is its rigorous diagnostic procedure, in which all ADHD diagnoses were carefully cross-validated by two board-certified physicians and confirmed using the K-SADS-E to ensure high diagnostic precision. Furthermore, to the best of our knowledge, this is the first study to specifically explore presentation-related associations between resting-state QEEG alpha activity and working memory performance in children with ADHD across EO and EC conditions. Future longitudinal studies, incorporating larger sex-balanced cohorts and typically developing controls, are warranted to replicate these electrophysiological–cognitive associations and further elucidate the neurophysiological mechanisms underlying cognitive deficits in ADHD.

## 5. Conclusions

Frontocentral alpha activity during the EO resting state was positively associated with working memory performance in children with ADHD-I, but not in those with ADHD-C. These findings highlight presentation-related differences in QEEG–cognition relationships and support the potential utility of EO resting-state alpha activity as an electrophysiological correlate of working memory performance in pediatric ADHD-I.

## Figures and Tables

**Figure 1:**
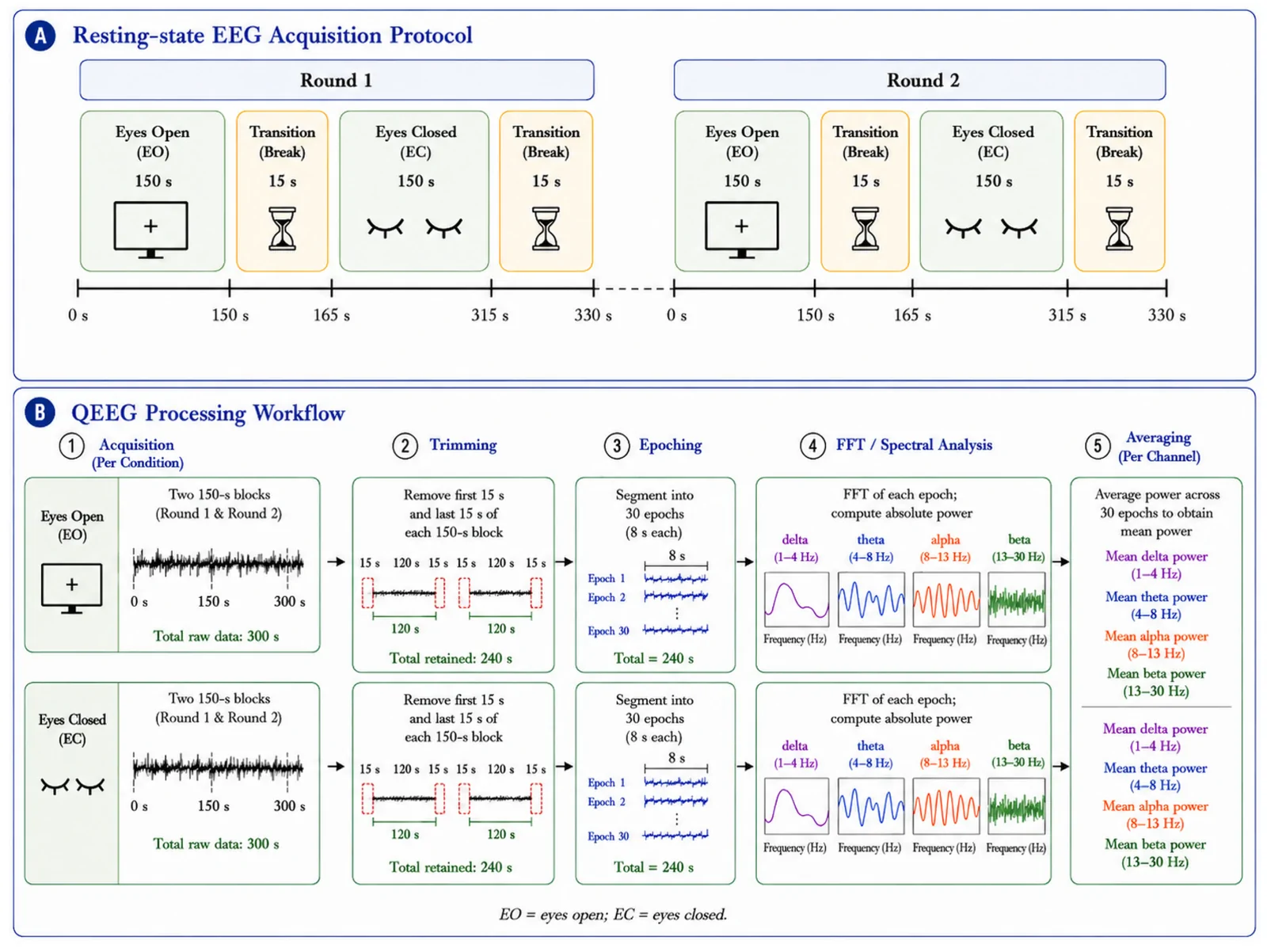
Experimental paradigm and quantitative electroencephalography (QEEG) analytical workflow: (**A**) Resting-state EEG acquisition protocol consisting of two identical rounds. Each round comprised a 150 s eyes-open period (EO; visual fixation on a black cross), a 15 s transition break, a 150 s eyes-closed period (EC), and a subsequent 15 s transition break. (**B**) Five-step QEEG processing pipeline: (1) acquisition of two 150 s EEG blocks per condition, yielding 300 s of raw data for each of the EO and EC conditions; (2) removal of the initial and final 15 s from each 150 s block to minimize transition-related artifacts and signal instability, leaving 240 s of retained data per condition; (3) segmentation of the retained data into 30 non-overlapping, artifact-free epochs of 8 s each (after Scan 4.5 and ICA-ADJUST artifact correction); (4) fast Fourier transform-based spectral analysis to calculate absolute power in four frequency bands: delta (1–4 Hz), theta (4–8 Hz), alpha (8–13 Hz), and beta (13–30 Hz); and (5) averaging of band-specific power across the 30 epochs to obtain the mean power for each EEG channel. The EEG waveforms and spectral traces are schematic illustrations and do not represent data from an individual participant.

**Figure 2:**
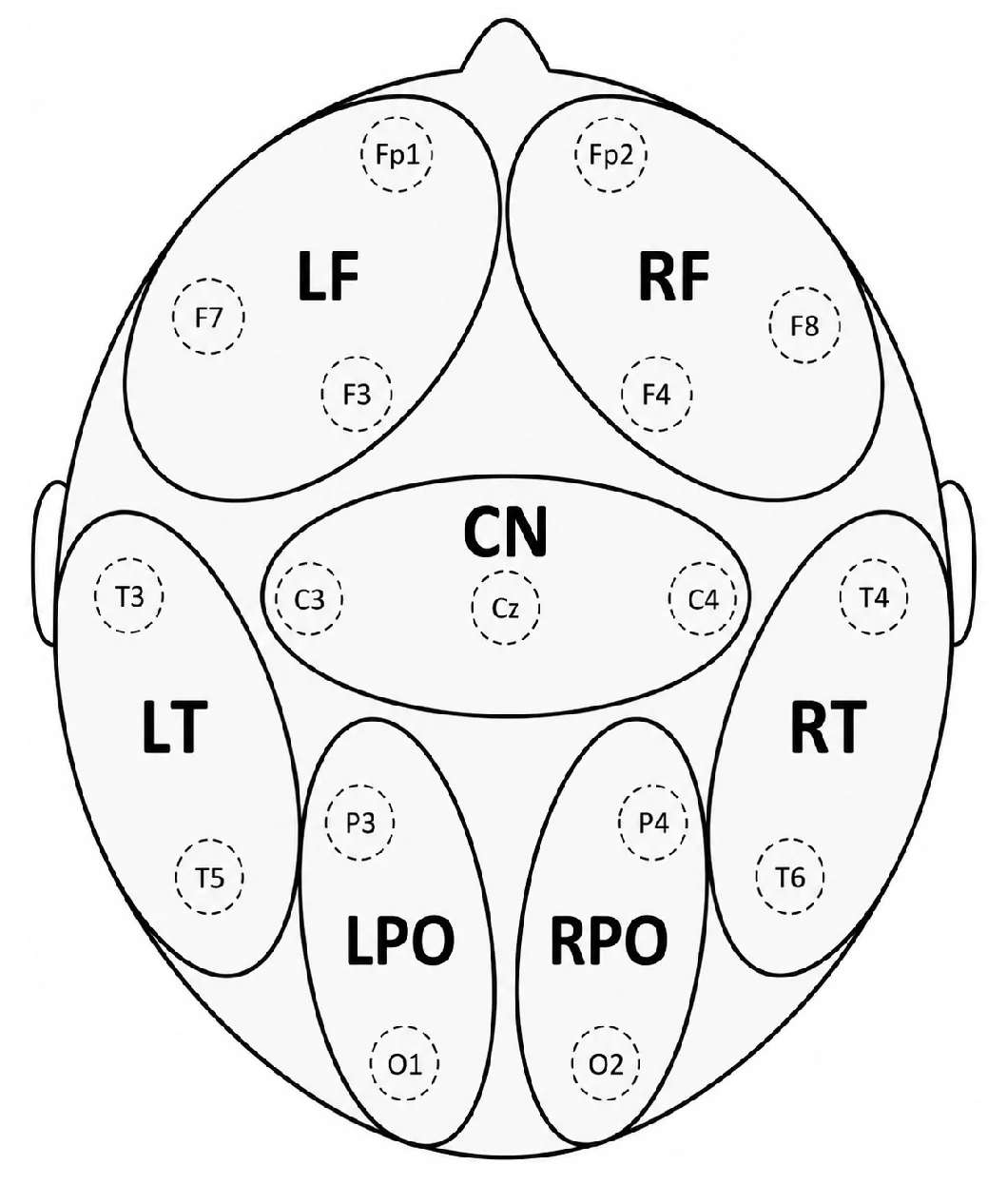
Electrodes are grouped into left frontal (LF), right frontal (RF), central (CN), left temporal (LT), right temporal (RT), left parieto-occipital (LPO), and right parieto-occipital (RPO) regions based on the international 10–20 system. Each region includes multiple electrodes (e.g., LF: Fp1, F3, and F7), and regional values were derived by averaging absolute power across all electrodes within each area. The top of the figure indicates the anterior direction.

**Figure 3:**
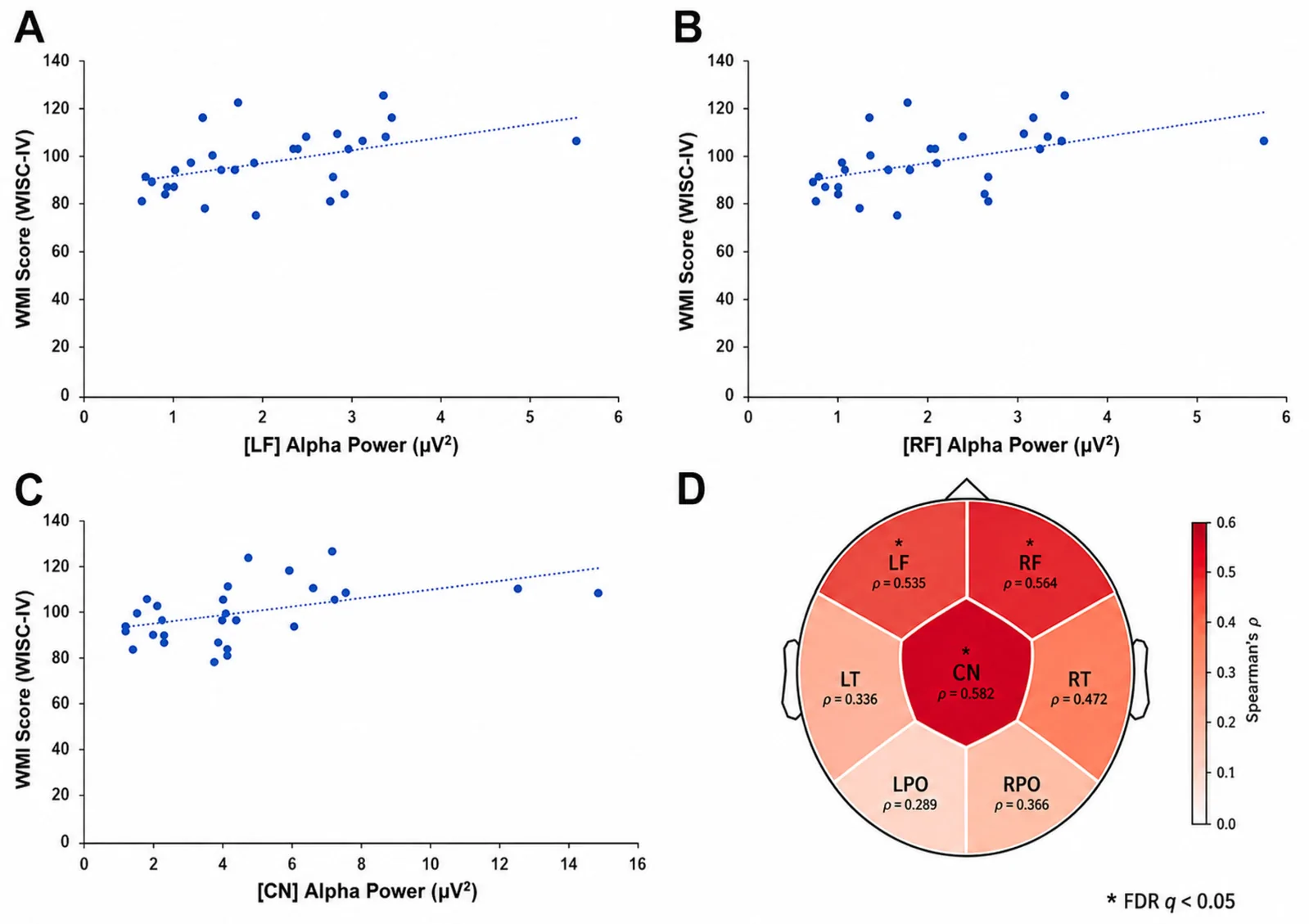
Correlations between resting-state eyes-open (EO) alpha power and Working Memory Index (WMI; WISC-IV) in the ADHD-I presentation (*n* = 29). Scatter plots show positive correlations between WMI scores and absolute alpha power in the (**A**) left frontal [LF], (**B**) right frontal [RF], and (**C**) central [CN] regions. (**D**) A schematic parcellation map illustrates the spatial distribution of Spearman’s *ρ*. Color intensity reflects correlation magnitude, and asterisks (*) denote regions surviving false discovery rate (FDR) correction (*q* < 0.05). Note: Dotted lines indicate simple linear trends for visual orientation only. Sensitivity analyses excluding potential high-power outliers did not materially alter the results. See Figure 2 for exact region-of-interest (ROI) electrode definitions.

**Table 1:** Demographic and clinical characteristics of the ADHD cohort and its presentations.

Characteristics	Total ADHD (*N* = 45)	ADHD-I (*n* = 29)	ADHD-C (*n* = 16)	*p*-Value
**Demographics**				
Age (years)	10.27 ± 1.97	10.59 ± 2.04	9.69 ± 1.74	0.145
Male sex, *n* (%)	36 (80.0)	21 (72.4)	15 (93.8)	0.127
BMI (kg/m^2^)	18.51 ± 4.21	18.85 ± 4.27	17.84 ± 4.17	0.479
**Birth History**				
Gestational Age (weeks)	37.69 ± 3.00	37.36 ± 3.33	38.36 ± 2.17	0.250
Birth Weight (g)	3088.5 ± 568.7	3070.5 ± 594.4	3124.5 ± 533.2	0.747
**Cognitive Profiles**				
Full-Scale IQ	102.24 ± 12.57	100.29 ± 11.04	106.14 ± 14.85	0.161
WMI	98.74 ± 14.46	97.36 ± 13.35	101.50 ± 16.64	0.478
PSI	96.55 ± 14.31	94.89 ± 14.50	99.86 ± 13.84	0.403

Note: Continuous variables are presented as mean ± standard deviation (SD) and were compared between the ADHD-I and ADHD-C presentations using the independent-samples *t*-test. Categorical variables are presented as numbers (percentages) and were compared using the chi-square test or Fisher’s exact test, as appropriate. Normality was assessed using the Shapiro–Wilk test. Abbreviations: ADHD-C, combined attention-deficit/hyperactivity disorder; ADHD-I, predominantly inattentive attention-deficit/hyperactivity disorder; BMI, body mass index; IQ, intelligence quotient; WMI, Working Memory Index; PSI, Processing Speed Index.

**Table 2:** Correlations of eyes-open alpha power with Working Memory Index in ADHD-I (*n* = 29).

Region of Interest (ROI)	Spearman’s *ρ* [95% CI]	Raw *p*-Value	FDR *q*-Value
Central (CN)	**0.582** [0.320, 0.729]	**0.001**	**0.014**
Right Frontal (RF)	**0.564** [0.249, 0.772]	**0.001**	**0.014**
Left Frontal (LF)	**0.535** [0.192, 0.783]	**0.003**	**0.028**
Right Temporal (RT)	0.472 [0.092, 0.728]	**0.010**	0.070
Right Parieto-Occipital (RPO)	0.366 [−0.041, 0.670]	0.051	0.285
Left Temporal (LT)	0.336 [−0.057, 0.686]	0.074	0.345
Left Parieto-Occipital (LPO)	0.289 [−0.106, 0.652]	0.128	0.512

Note: *ρ* represents Spearman’s rank correlation coefficient. Alpha power was recorded in the eyes-open resting state. 95% CIs were estimated using 1000 bootstrap resamples. To control for the false discovery rate (FDR), the Benjamini–Hochberg procedure was applied across a family of 28 comparisons (four frequency bands × seven regions of interest [ROIs]). Statistically significant values (*p* < 0.05, FDR *q* < 0.05) and their corresponding correlation coefficients are highlighted in bold.

**Table 3:** General Linear Model (GLM) analyses testing Presentation × WMI interaction on eyes-open alpha power (*N* = 45).

Region of Interest	Model *R*^2^	Interaction *F* (1, 41)	Interaction *p*	Slope Difference Δ*B* [95% CI]
Central (CN)	0.253	8.314	**0.006**	+0.148 [0.044, 0.252]
Right Frontal (RF)	0.194	4.804	**0.034**	+0.043 [0.003, 0.083]
Left Frontal (LF)	0.153	4.790	**0.035**	+0.044 [0.003, 0.085]

Note: *N* = 45 (ADHD-I: *n* = 29; ADHD-C: *n* = 16). Dependent variable: absolute alpha power (μV^2^) during the resting-state eyes-open (EO) condition. ADHD-C served as the reference group in parameter estimation. Δ*B* denotes the steeper positive WMI–alpha slope in ADHD-I relative to ADHD-C. Abbreviations: CI, confidence interval; WMI, Working Memory Index. Bold values indicate *p* < 0.05.

## Data Availability

The data presented in this study is available on request from the corresponding author. The data are not publicly available due to institutional, privacy, and ethical restrictions related to pediatric clinical data.

## Supplementary Materials

The following supporting information can be downloaded at https://www.mdpi.com/article/10.3390/diagnostics16193104/s1, Table S1: Spearman’s correlations between resting-state alpha power and Working Memory Index (WMI) across ADHD presentations; Table S2: Spearman’s correlations between resting-state alpha power and Processing Speed Index (PSI) across ADHD presentations; Table S3: Spearman’s correlations between resting-state delta, theta, and beta powers and Working Memory Index (WMI) across ADHD presentations; Table S4: Spearman’s correlations between resting-state delta, theta, and beta powers and Processing Speed Index (PSI) across ADHD presentations; and Table S5: Pharmacokinetic properties and clearance profiles of ADHD medications in the study cohort (*N* = 45).

## Abbreviations

The following abbreviations are used in this manuscript:

ADHD	Attention-deficit/hyperactivity disorder
ADHD-I	Predominantly inattentive ADHD
ADHD-C	Combined ADHD
ADHD-HI	Predominantly hyperactive/impulsive presentation
BP	Band power
CAST	Childhood Autism Spectrum Test
CN	Central
CNS	Central nervous system
CI	Confidence interval
DSM-5	Diagnostic and Statistical Manual of Mental Disorders, 5th edition
EC	Eyes closed
EO	Eyes open
EOG	Electrooculography
EEG	Electroencephalography
EMG	Electromyography/electromyographic
FDR	False discovery rate
FFT	Fast Fourier transform
GLMs	General Linear Models
ICA	Independent component analysis
IQ	Intelligence quotient
IAF	Individual alpha frequency
IRB	Institutional Review Board
K-SADS-E	Kiddie Schedule for Affective Disorders and Schizophrenia—Epidemiologic Version
LF	Left frontal
LPO	Left parieto-occipital
LT	Left temporal
PSI	Processing Speed Index
QEEG	Quantitative electroencephalography
RF	Right frontal
ROIs	Regions of interest
RPO	Right parieto-occipital
RT	Right temporal
SD	Standard deviation
TD	Typically developing
WISC	Wechsler Intelligence Scale for Children
WISC-IV	Wechsler Intelligence Scale for Children, Fourth Edition
WMI	Working Memory Index
