# Supplementary material for: Eyes-Open Fronto-Central Alpha Activity as an Electrophysiological Correlate of Working Memory in Children with Predominantly Inattentive ADHD
Source: Diagnostics (Basel). 2026 Sep 24;16(19):3104. doi: 10.3390/diagnostics16193104 (PMC13664443; doi:10.3390/diagnostics16193104)
Supplement: Supplementary file 1 [file diagnostics-16-03104-s001.zip › Supplementary Table S1.pdf]

Table S1. Spearman's correlations between resting-state alpha power and Working Memory Index (WMI) across ADHD presentations.

**State & Region of Interest (ROI)**	**Total ADHD (N = 45)**	**ADHD-I (n = 29)**	**ADHD-C (n = 16)**
**Eyes-Open (EO)**			
Left Frontal (LF)	0.300	**0.535**	−0.102
Right Frontal (RF)	0.367	**0.564**	0.016
Central (CN)	0.276	**0.582**	−0.360
Left Temporal (LT)	0.190	0.336	−0.027
Right Temporal (RT)	0.302	0.472	−0.025
Left Parieto-Occipital (LPO)	0.147	0.289	−0.114
Right Parieto-Occipital (RPO)	0.130	0.366	−0.158
**Eyes-Closed (EC)**			
Left Frontal (LF)	0.217	0.192	0.105
Right Frontal (RF)	0.266	0.274	0.127
Central (CN)	0.050	0.074	−0.231
Left Temporal (LT)	0.027	0.138	−0.317
Right Temporal (RT)	0.172	0.266	−0.193
Left Parieto-Occipital (LPO)	0.031	0.094	−0.311
Right Parieto-Occipital (RPO)	0.050	0.136	−0.264

Notes: Values represent Spearman's rank correlation coefficients (ρ). Bold values indicate correlations that remained statistically significant after rigorous false discovery rate (FDR) correction (q<0.05) applying the Benjamini-Hochberg procedure across a predefined family of 28 comparisons (4 frequency bands × 7 ROIs). Abbreviations: ADHD-C, combined attention-deficit/hyperactivity disorder; ADHD-I, predominantly inattentive attention-deficit/hyperactivity disorder
