# Supplementary material for: Eyes-Open Fronto-Central Alpha Activity as an Electrophysiological Correlate of Working Memory in Children with Predominantly Inattentive ADHD
Source: Diagnostics (Basel). 2026 Sep 24;16(19):3104. doi: 10.3390/diagnostics16193104 (PMC13664443; doi:10.3390/diagnostics16193104)
Supplement: Supplementary file 1 [file diagnostics-16-03104-s001.zip › Supplementary Table S2.pdf]

Table S2. Spearman's correlations between resting-state alpha power and Processing Speed Index (PSI) across ADHD presentations.

State & Region of Interest (ROI)	Total ADHD (N = 45)	ADHD-I (n = 29)	ADHD-C (n = 16)
**Eyes-Open (EO)**			
Left Frontal (LF)	0.090	0.249	−0.147
Right Frontal (RF)	0.160	0.264	−0.068
Central (CN)	−0.005	0.218	−0.409
Left Temporal (LT)	0.085	0.143	0.039
Right Temporal (RT)	0.165	0.205	0.280
Left Parieto-Occipital (LPO)	0.120	0.172	0.058
Right Parieto-Occipital (RPO)	0.065	0.155	−0.025
**Eyes-Closed (EC)**			
Left Frontal (LF)	0.102	0.009	0.322
Right Frontal (RF)	0.107	0.040	−0.094
Central (CN)	−0.096	−0.129	−0.129
Left Temporal (LT)	−0.038	0.003	0.336
Right Temporal (RT)	−0.044	−0.136	0.094
Left Parieto-Occipital (LPO)	−0.066	−0.095	−0.008
Right Parieto-Occipital (RPO)	−0.110	−0.175	0.000

Notes: Values represent Spearman's rank correlation coefficients (ρ). The Benjamini-Hochberg false discovery rate (FDR) procedure was applied across a predefined family of 28 comparisons (4 frequency bands × 7 ROIs) for each state and subgroup. No correlations achieved statistical significance after FDR correction (q<0.05). Abbreviations: ADHD-C, combined attention-deficit/hyperactivity disorder; ADHD-I, predominantly inattentive attention-deficit/hyperactivity disorder.
