# Supplementary material for: Eyes-Open Fronto-Central Alpha Activity as an Electrophysiological Correlate of Working Memory in Children with Predominantly Inattentive ADHD
Source: Diagnostics (Basel). 2026 Sep 24;16(19):3104. doi: 10.3390/diagnostics16193104 (PMC13664443; doi:10.3390/diagnostics16193104)
Supplement: Supplementary file 1 [file diagnostics-16-03104-s001.zip › Supplementary Table S3.pdf]

Table S3. Spearman's correlations between resting-state delta, theta, and beta powers and Working Memory Index (WMI) across ADHD presentations.

Frequency Band	Region of Interest (ROI)	Total ADHD ($N = 45$)	ADHD-I ($n = 29$)	ADHD-C ($n = 16$)
	**Eyes-Open (EO)**			
**Delta (1–4 Hz)**	Left Frontal (LF)	−0.037	0.160	−0.351
	Right Frontal (RF)	−0.008	0.225	−0.407
	Central (CN)	0.080	0.322	−0.263
	Left Temporal (LT)	0.118	0.376	−0.253
	Right Temporal (RT)	0.316	0.523	−0.090
	Left Parieto-Occipital (LPO)	0.148	0.445	−0.248
	Right Parieto-Occipital (RPO)	0.257	0.486	−0.258
**Theta (4–8 Hz)**	Left Frontal (LF)	0.207	0.419	−0.220
	Right Frontal (RF)	0.219	0.481	−0.367
	Central (CN)	0.299	0.486	−0.016
	Left Temporal (LT)	0.262	0.457	−0.152
	Right Temporal (RT)	0.368	0.575	−0.059
	Left Parieto-Occipital (LPO)	0.254	0.502	−0.127
	Right Parieto-Occipital (RPO)	0.291	0.479	−0.041
**Beta (13–30 Hz)**	Left Frontal (LF)	0.086	0.399	−0.441
	Right Frontal (RF)	0.140	0.369	−0.351
	Central (CN)	0.180	0.487	−0.382
	Left Temporal (LT)	−0.159	0.080	−0.466
	Right Temporal (RT)	−0.056	0.181	−0.548
	Left Parieto-Occipital (LPO)	0.095	0.196	−0.125
	Right Parieto-Occipital (RPO)	0.147	0.285	−0.120
	**Eyes-Closed (EC)**			
**Delta (1–4 Hz)**	Left Frontal (LF)	−0.038	−0.110	0.129
	Right Frontal (RF)	0.003	−0.035	0.094
	Central (CN)	0.046	0.032	0.017
	Left Temporal (LT)	0.152	0.037	0.311
	Right Temporal (RT)	0.352	0.290	0.477
	Left Parieto-Occipital (LPO)	0.188	0.112	0.229
	Right Parieto-Occipital (RPO)	0.173	0.091	0.325
**Theta (4–8 Hz)**	Left Frontal (LF)	0.070	−0.053	0.303

	Right Frontal (RF)	0.086	−0.030	0.391
	Central (CN)	0.055	−0.070	0.262
	Left Temporal (LT)	0.169	0.004	0.383
	Right Temporal (RT)	0.221	0.115	0.402
	Left Parieto-Occipital (LPO)	0.096	−0.049	0.229
	Right Parieto-Occipital (RPO)	0.125	−0.011	0.300
**Beta (13–30 Hz)**	Left Frontal (LF)	0.165	0.094	0.430
	Right Frontal (RF)	0.166	0.069	0.488
	Central (CN)	0.058	0.054	0.085
	Left Temporal (LT)	0.084	0.097	0.096
	Right Temporal (RT)	0.010	−0.039	0.096
	Left Parieto-Occipital (LPO)	−0.176	−0.280	−0.008
	Right Parieto-Occipital (RPO)	−0.161	−0.249	−0.121

Notes: Values represent Spearman's rank correlation coefficients (ρ). To align with the primary theoretical focus of the present study on alpha-band oscillations (as detailed in the main text), statistical significance levels for other frequency bands are not highlighted here. These comprehensive matrices are provided solely for full data transparency. Abbreviations: ADHD-C, combined attention-deficit/hyperactivity disorder; ADHD-I, predominantly inattentive attention-deficit/hyperactivity disorder.
