# Supplementary material for: Eyes-Open Fronto-Central Alpha Activity as an Electrophysiological Correlate of Working Memory in Children with Predominantly Inattentive ADHD
Source: Diagnostics (Basel). 2026 Sep 24;16(19):3104. doi: 10.3390/diagnostics16193104 (PMC13664443; doi:10.3390/diagnostics16193104)
Supplement: Supplementary file 1 [file diagnostics-16-03104-s001.zip › Supplementary Table S4.pdf]

Table S4. Spearman's correlations between resting-state delta, theta, and beta powers and Processing Speed Index (PSI) across ADHD presentations.

Frequency Band	Region of Interest (ROI)	Total ADHD (N = 45)	ADHD-I (n = 29)	ADHD-C (n = 16)
	**Eyes-Open (EO)**			
**Delta (1–4 Hz)**	Left Frontal (LF)	−0.147	0.022	−0.485
	Right Frontal (RF)	−0.176	−0.044	−0.449
	Central (CN)	−0.261	−0.106	−0.470
	Left Temporal (LT)	−0.120	−0.037	−0.196
	Right Temporal (RT)	−0.142	−0.041	−0.449
	Left Parieto-Occipital (LPO)	−0.173	0.010	−0.452
	Right Parieto-Occipital (RPO)	−0.130	−0.008	−0.370
**Theta (4–8 Hz)**	Left Frontal (LF)	−0.048	0.198	−0.504
	Right Frontal (RF)	−0.006	0.218	−0.503
	Central (CN)	−0.112	0.093	−0.526
	Left Temporal (LT)	−0.028	0.117	−0.327
	Right Temporal (RT)	−0.029	0.175	−0.478
	Left Parieto-Occipital (LPO)	−0.083	0.134	−0.571
	Right Parieto-Occipital (RPO)	−0.083	0.152	−0.560
**Beta (13–30 Hz)**	Left Frontal (LF)	−0.021	0.194	−0.452
	Right Frontal (RF)	−0.112	0.118	−0.566
	Central (CN)	−0.121	−0.038	−0.316
	Left Temporal (LT)	−0.135	0.028	−0.359
	Right Temporal (RT)	−0.070	0.114	−0.483
	Left Parieto-Occipital (LPO)	−0.274	−0.217	−0.379
	Right Parieto-Occipital (RPO)	−0.258	−0.126	−0.445
	**Eyes-Closed (EC)**			
**Delta (1–4 Hz)**	Left Frontal (LF)	−0.064	−0.069	−0.088
	Right Frontal (RF)	−0.104	−0.116	−0.138
	Central (CN)	−0.180	−0.151	−0.146
	Left Temporal (LT)	−0.035	−0.132	0.196
	Right Temporal (RT)	−0.123	−0.103	−0.320
	Left Parieto-Occipital (LPO)	−0.136	−0.149	−0.143
	Right Parieto-Occipital (RPO)	−0.119	−0.099	−0.176
**Theta (4–8 Hz)**	Left Frontal (LF)	0.027	0.010	0.094

	Right Frontal (RF)	0.029	0.013	0.044
	Central (CN)	−0.057	−0.032	−0.107
	Left Temporal (LT)	−0.041	−0.020	−0.097
	Right Temporal (RT)	−0.095	−0.077	−0.300
	Left Parieto-Occipital (LPO)	−0.065	−0.002	−0.264
	Right Parieto-Occipital (RPO)	−0.090	−0.029	−0.287
**Beta (13–30 Hz)**	Left Frontal (LF)	−0.261	−0.274	−0.185
	Right Frontal (RF)	−0.231	−0.316	−0.030
	Central (CN)	−0.311	−0.383	−0.127
	Left Temporal (LT)	−0.205	−0.206	−0.220
	Right Temporal (RT)	−0.280	−0.382	−0.099
	Left Parieto-Occipital (LPO)	−0.212	−0.371	0.146
	Right Parieto-Occipital (RPO)	−0.260	−0.262	−0.052

Notes: Values represent Spearman's rank correlation coefficients (ρ). To align with the primary theoretical focus of the present study on alpha-band oscillations (as detailed in the main text), statistical significance levels for other frequency bands are not highlighted here. These comprehensive matrices are provided solely for full data transparency. Abbreviations: ADHD-C, combined attention-deficit/hyperactivity disorder; ADHD-I, predominantly inattentive attention-deficit/hyperactivity disorder.
