# Supplementary material for: Eyes-Open Fronto-Central Alpha Activity as an Electrophysiological Correlate of Working Memory in Children with Predominantly Inattentive ADHD
Source: Diagnostics (Basel). 2026 Sep 24;16(19):3104. doi: 10.3390/diagnostics16193104 (PMC13664443; doi:10.3390/diagnostics16193104)
Supplement: Supplementary file 1 [file diagnostics-16-03104-s001.zip › Supplementary Table S5.pdf]

Table S5. Pharmacokinetic properties and clearance profiles of ADHD medications in the study cohort ($N = 45$).

Formulation	Patients, *n* (%)	Elimination Half-life (t1/2, h)	Estimated Clearance after 24-h Washout
**No medication use**	7 (15.6%)	—	—
**Ritalin®**	7 (15.6%)	2.0 – 2.5	>99.9%
**Ritalin LA®**	4 (8.9%)	2.4 – 2.5	>99.9%
**Concerta®**	19 (42.2%)	3.5	99.1%
**Concerta® + Ritalin®**	7 (15.6%)	2.0 – 3.5	99.1% – >99.9%
**Strattera®**	1 (2.2%)	5.2	95.9%

Notes: Reference sources for pharmacokinetic parameters and formulation clearance criteria: Drug package inserts (Ritalin®, Ritalin LA®, Concerta®, and Strattera®) and the Micromedex database. Estimated Clearance after 24-h Washout = $[1 - 0.5^{(24/t1/2)}] \times 100\%$, Abbreviations: ADHD, attention-deficit/hyperactivity disorder
